# Incidental diagnosis of intraductal papillary neoplasm of the bile duct: Imaging findings from a case report

**DOI:** 10.1016/j.radcr.2025.06.008

**Published:** 2025-06-27

**Authors:** Imene Chafai, Alba Igual-Rouilleault, Morgane Van Wettere

**Affiliations:** a Department of Radiology, Erasme University Hospital, Brussels, Belgium; b Department of Abdominal Radiology, Cliniques Universitaires Saint-Luc, Brussels, Belgium

**Keywords:** IPNB, Bile duct, MRI, Cholangiocarcinoma

## Abstract

Intraductal papillary neoplasms of the bile ducts (IPNBs) represent a rare biliary tract tumors with malignant potential, requiring surgical resection to prevent progression to cholangiocarcinoma. In this case, an 83-year-old woman with an obstructive pattern in liver function tests was incidentally found to have a solid mass in the left hepatic lobe on ultrasound. Liver MRI suggested a polypoid intraductal papillary neoplasm of the left bile duct, and histopathological analysis after surgical resection confirmed low-grade dysplasia with a pancreaticobiliary subtype. Postoperative MRI showed no residual tumor, and the patient remains under close observation with no evidence of recurrence. This case highlights the importance of imaging techniques in the early detection and characterization of IPNB, and includes a summary of its morphological subtypes.

## Introduction

Intraductal papillary neoplasms of the bile duct (IPNB) are a rare entity characterized by intraluminal papillary growths that can affect both intrahepatic and extrahepatic bile ducts [1]. First described in 2001 by Chen et al. [2], IPNB remain a relatively recent disease, officially recognized as a bile duct tumor by the World Health Organization (WHO) in 2010 [3]. It accounts for only 5% to 15% of all bile duct tumors, with a higher prevalence in East Asia [4]. IPNB typically appear in the sixth decade of life, with a male predominance [5]. Clinical symptoms and laboratory findings are often similar to those seen in obstructive biliary diseases, including intermittent right hypochondrial pain, jaundice, elevated liver enzyme levels, and, in some cases, cholangitis [6]. However, studies indicate that 5% to 29% of patients may remain asymptomatic [7]. Elevated serum tumor markers, particularly carcinoembryonic antigen (CEA) and carbohydrate antigen (CA19-9), are detected in approximately 25% and 40% of cases, respectively [8]. Diagnosis primarily relies on magnetic resonance imaging (MRI) and cholangioscopy, as conventional imaging techniques, such as computed tomography (CT) and ultrasonography (US), may underestimate superficial extension and tumor progression, particularly for small papillary lesions [9]. Because it can progress to cholangiocarcinoma, surgical resection is the treatment of choice.

## Case report

An asymptomatic 83-year-old French woman with no history of prior surgeries was incidentally found to have an abnormal obstructive pattern in liver function tests (LFTs) during a routine cholesterol check. Other laboratory tests, including a complete blood count and inflammatory markers, were within normal limits, with no signs of infection or systemic involvement. Physical examination revealed no abnormalities, and the patient was clinically stable.

**Fig. 1:**
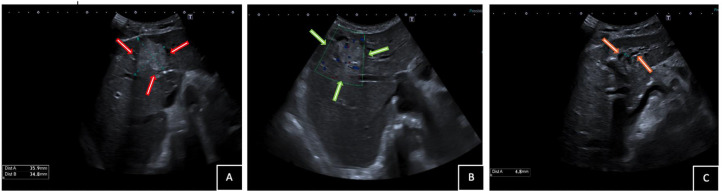
Abdominal ultrasound images show (A) a hyperechoic solid mass of 36 mm in the left hepatic lobe (red arrows) with (B) mild vascularization on Doppler (green arrows) causing (C) left intrahepatic ductal dilatation (orange arrows).

**Fig. 2:**
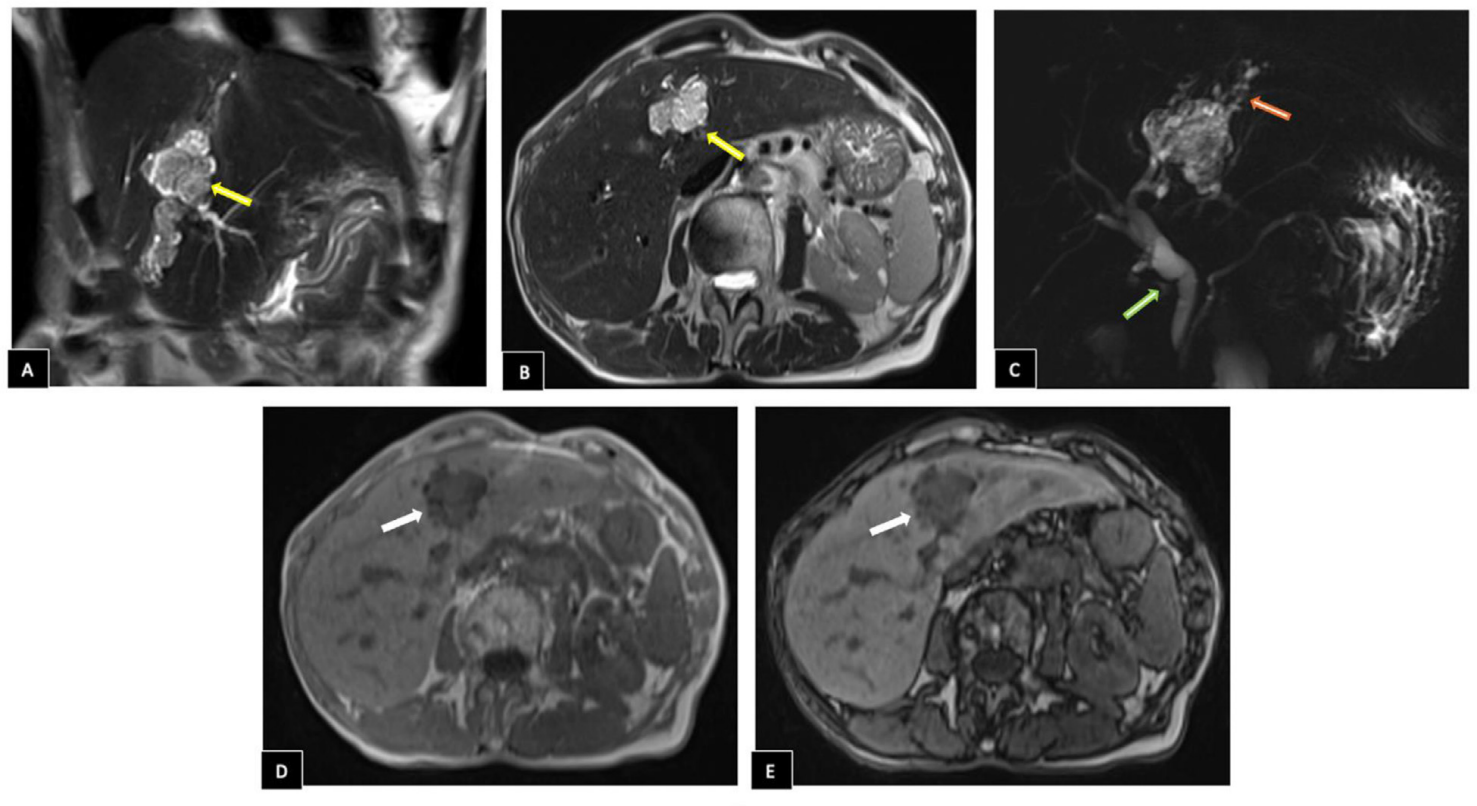
Liver MRI Imaging Findings. (A, B) Coronal and axial T2 images revealing an intraductal mass between segments IV and III with lobulated contours heterogeneous hyperintense, extending within the biliary duct of segment III (yellow arrows). (C) Coronal conventional T2-weighted MR cholangiography shows mild upstream intrahepatic segmental bile duct dilatation (red arrow ) as well as moderate dilatation of extrahepatic bile ducts (green arrow). (D) In- and (e) opposed-phase T1-weighted imaging indicate absence of intralesional fat (white arrows).

As part of the diagnostic, an abdominal ultrasound was performed, revealing a 36 mm hyperechoic solid mass in the left hepatic lobe with mild upstream intrahepatic ductal dilatation (Fig. 1). CEA and CA 19-9 levels were within normal ranges, measuring 1.8 ng/mL and <9 U/mL, respectively. A liver MRI was performed for further characterization, revealing an intraductal mass located between segments IV and III. The lesion appeared hypointense on T1-weighted sequences relative to the adjacent hepatic parenchyma and displayed a heterogeneous hyperintense signal on T2-weighted sequences. In- and opposed-phase T1-weighted imaging confirmed the absence of intralesional fat. Coronal conventional T2-weighted MR cholangiography showed mild upstream intrahepatic segmental bile duct dilatation due to a tumoral mass effect, accompanied by moderate extrahepatic bile duct dilatation, likely secondary to excessive tumor mucin production (Fig. 2).

**Fig. 3:**
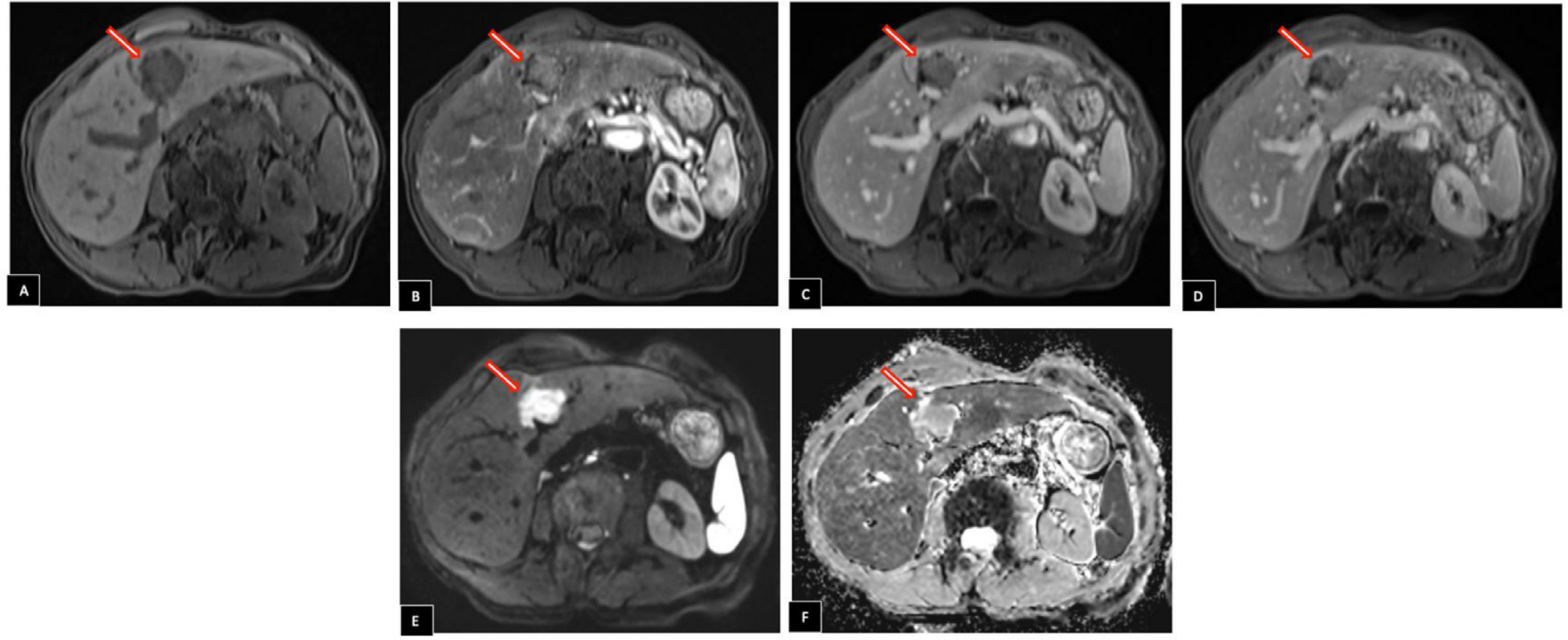
Liver MRI Imaging Findings. (A) Before contrast injection, the mass appears hypointense on T1-weighted imaging. (B) After contrast administration, it demonstrates arterial phase enhancement, which (C, D) persists slightly in the venous phases. (E) Axial diffusion-weighted imaging shows high signal intensity in the mass with (F) corresponding low signal intensity on the apparent diffusion coefficient (ADC) map. The mass and its characteristics are indicated by the arrows.

Following contrast administration, the mass showed arterial phase enhancement, with slight persistence in the venous phases. On axial diffusion-weighted imaging, it presented high signal intensity with corresponding low signal intensity on the apparent diffusion coefficient (ADC) map (Fig. 3).

**Fig. 4:**
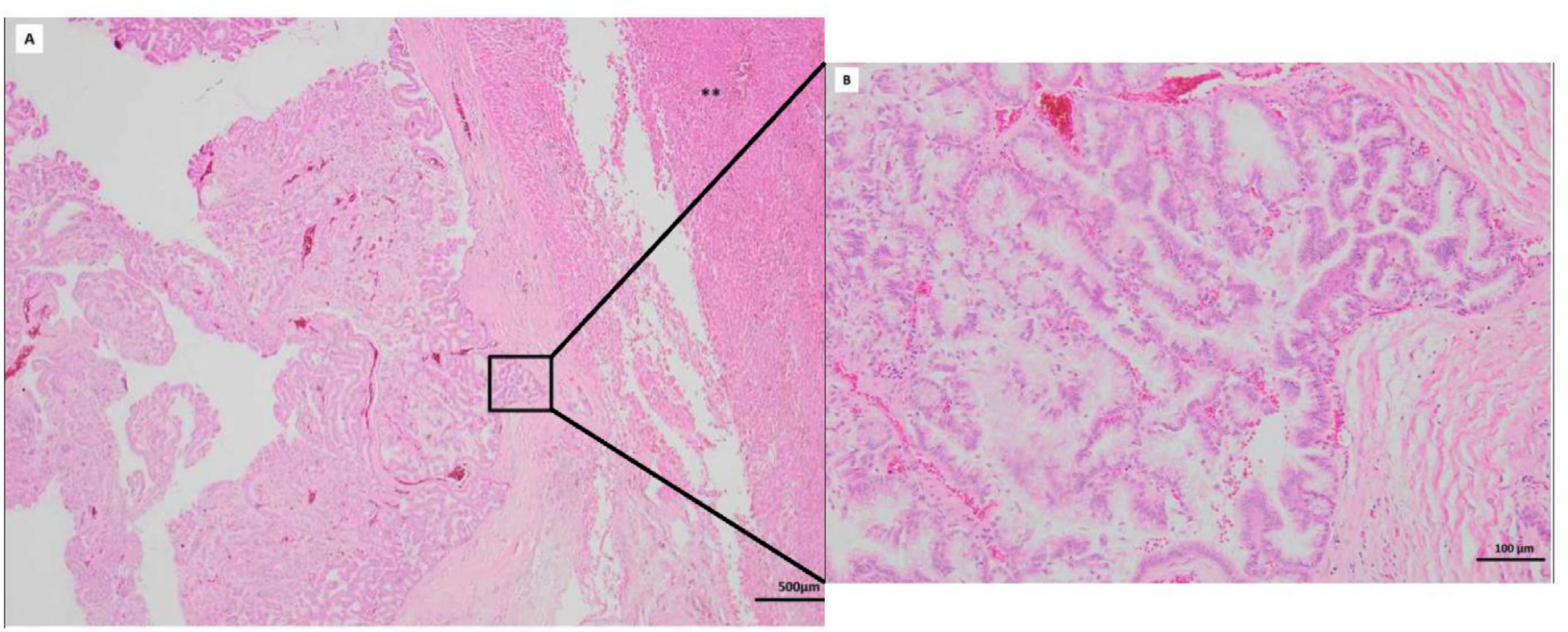
Histopathological examination of the surgical specimen. (A) Low magnification view shows an intraductal papillary lesion (quadrate). Asterisks mark the normal liver adjacent to the lesion. (B) Higher magnification view demonstrates an epithelium composed of cylindrical cells maintaining epithelial polarity with subtle nuclear-cytoplasmic atypia (small nucleoli).

The case was reviewed by the hepatic oncologic multidisciplinary team, which decided surgical treatment. One month later, the patient underwent an extended left hemi-hepatectomy and cholecystectomy. Histopathological examination of hematoxylin and eosin (H&E) stained slides (Fig. 4) confirmed a low-grade dysplasia and a pancreaticobiliary IPNB subtype. The neoplasm was confined to the biliary tract (pT1) with no lymph node involvement.

**Fig. 5:**
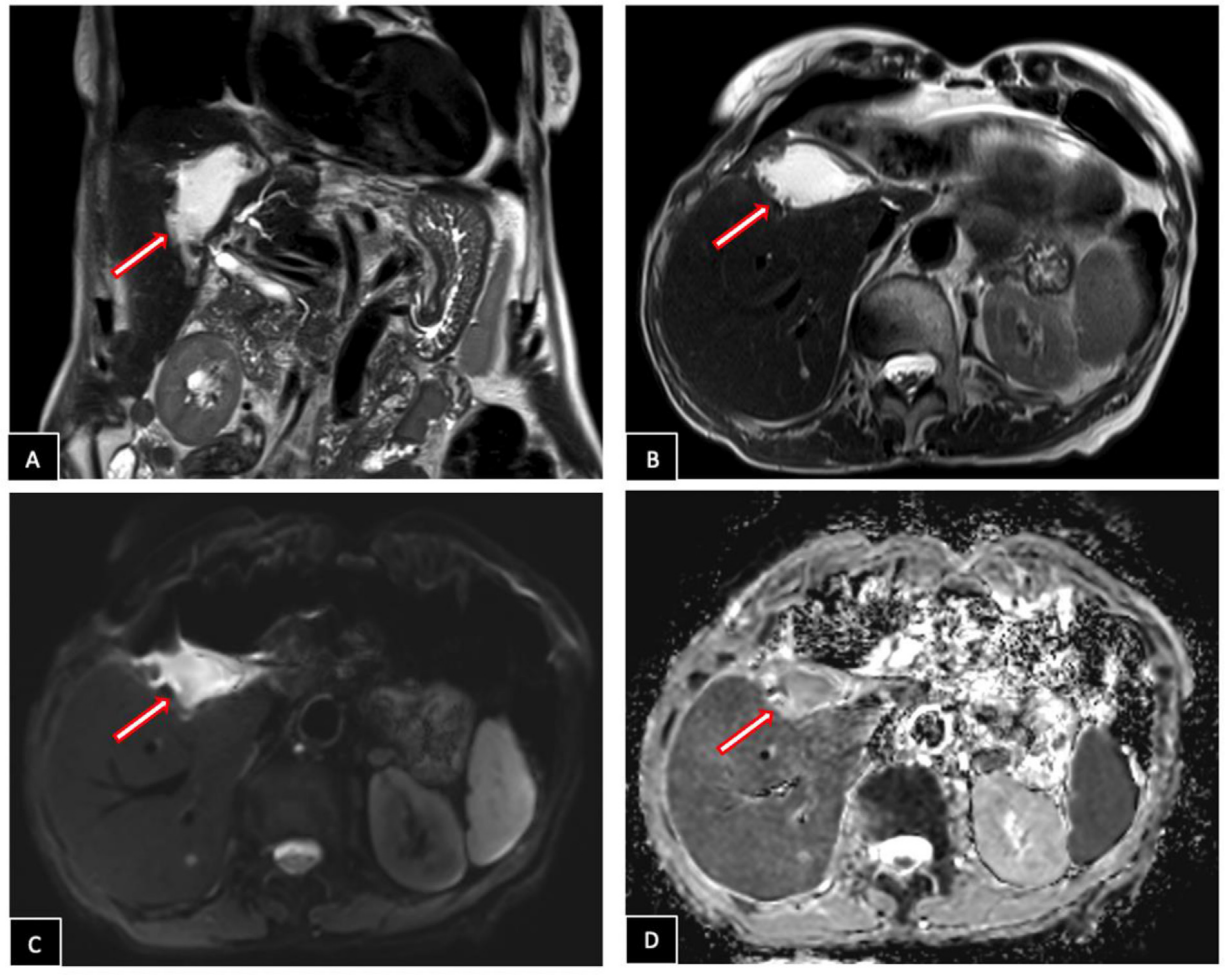
Post-operative hepatic MRI. Well-shaped area at the site of surgical resection, hyperintense on T2-weighted images (A, B) with restricted diffusion (C, D), corresponding to hemostatic material (arrows).

**Fig. 6:**
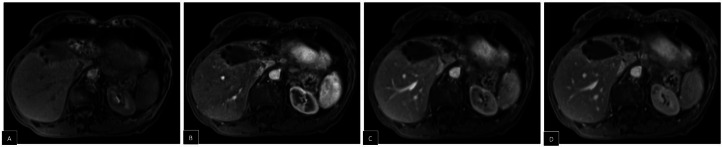
Post-operative hepatic MRI. T1-weighted precontrast image (A) and post-contrast images in arterial (B), portal (C) and delayed (D) phases reveal an absence of invasive or pathologic contrast enhancement suggesting no residual tumor.

Post-operative hepatic MRI showed hemostatic material at the resection site, with no residual tumor or masses detected (Fig. 5, Fig. 6). The patient recovered uneventfully and was discharged home 3 days after surgery.

## Discussion

Cholangiocarcinoma (CCA) arises from the biliary epithelium and is often preceded by well-defined premalignant lesions. The two main precursor lesions are biliary intraepithelial neoplasia (BilIN) and intraductal papillary neoplasm of the bile duct (IPNB) [10]. While BilIN is a microscopic lesion detectable only through histopathological analysis, IPNB is a macroscopically visible entity that can be identified on imaging studies.

IPNB present four different subtypes defined by morphological appearances [4]. The polypoid form is the most common and appears as an intraductal, cauliflower-like mass, sometimes reaching significant dimensions, as in our case. Upstream bile duct dilatation may occur due to a mass-induced mechanical obstruction. T2-weighted MRI is particularly useful in differentiating this subtype, as it provides high contrast between the luminal bile and the neoplasm. Unlike cholangiocarcinoma, IPNB in this form exhibits limited fibrous stroma and a fibrovascular stalk, making delayed-phase enhancement rare.

Another subtype, known as the superficial/mucosal spreading type, grows parallel to the bile duct wall, making it more difficult to detect on imaging. It is frequently associated with excessive mucin production, leading to lobar or segmental bile duct dilatation without a clearly visible obstructing mass. In long-standing cases, this presentation can lead to atrophy of the affected hepatic lobe or segment due to chronic obstruction.

In some cases, IPNB adopts a cast-like intraductal pattern, where it fills the bile duct lumen over a variable length. On ultrasound, this subtype appears as uniformly echogenic duct walls, while CT and MRI reveal enhancing intraluminal masses. This pattern leads to ductal narrowing without invasion of periductal tissues unless malignant invasion occurs. Due to its imaging similarities with primary sclerosing cholangitis or fascioliasis, histological evaluation is often necessary to establish a definitive diagnosis.

A final variation, the cystic subtype, results from excessive mucin production, leading to focal cystic dilatation of the bile ducts (pseudocyst pattern). Imaging reveals uni- or multilocular cystic lesions, and occasionally exhibit mural nodules. This form requires differentiation from mucinous cystic neoplasm (MCN), which typically does not communicate with the bile ducts [11].

Histologically, IPNB is classified into 4 phenotypes based on hematoxylin and eosin staining: pancreatobiliary (PB), intestinal, gastric, and oncocytic. The PB and intestinal patterns are the most prevalent and often associated with invasive lesions. The gastric and oncocytic subtypes, although less common, generally exhibit a more indolent clinical course [12].

IPNB predominantly occurs in an intrahepatic or hilar location [13]. The majority of intrahepatic IPNBs are found in the left-sided biliary ducts, although the exact reason for this localization remains unclear [14]. Risk factors for IPNB include hepatolithiasis, liver parasitic infections (such as *Clonorchis sinensis* and *Opisthorchis viverrini*), primary sclerosing cholangitis, biliary malformations—including choledochal cysts—and genetic conditions such as familial adenomatous polyposis or Gardner syndrome [15].

The anatomical location and geographical distribution of IPNBs have been linked to varying risks of stromal invasion. Tumors originating in the extrahepatic bile ducts and affecting Caucasian patients typically exhibit higher rates of stromal invasion. In contrast, intrahepatic IPNBs and those occurring in Asian populations often follow a more indolent course [16].

Lee et al. [9] identified key MRI features that help distinguish IPNBs with invasive carcinoma from noninvasive forms. These features include an intraductal visible mass, tumor size ≥ 2.5 cm, multifocal involvement, adjacent organ invasion, and bile duct wall thickening.

IPNB has a better prognosis and surgical outcomes compared to conventional cholangiocarcinoma. However, due to its premalignant nature, surgery with a radical intent remains the preferred treatment. Endoscopic resection is currently considered for patients unfit for surgery. Despite the potential for cure after excision with negative margins, patients diagnosed with IPNB require close monitoring for recurrence or the development of other pancreatic-biliary neoplasms.

## Conclusion

IPNBs are preinvasive neoplasms of the bile duct with high malignant potential and a tendency to progress to invasive CCA. Radiologists play a crucial role in diagnosing IPNB, whether as an incidental finding or in symptomatic patients. Given their complexity, increasing awareness of imaging features is essential for accurate diagnosis and precise characterization.

## Patient consent

We declare that written informed consent for the publication of this case report and accompanying images was obtained from the patient, and a signed document is in our possession.
